# Moral Foundations Theory Among Autistic and Neurotypical Children

**DOI:** 10.3389/fpsyg.2021.782610

**Published:** 2022-01-14

**Authors:** Erin Elizabeth Dempsey, Chris Moore, Shannon A. Johnson, Sherry H. Stewart, Isabel M. Smith

**Affiliations:** ^1^Department of Psychology and Neuroscience, Dalhousie University, Halifax, NS, Canada; ^2^Department of Psychiatry, Dalhousie University, Halifax, NS, Canada; ^3^Department of Pediatrics, Dalhousie University, Halifax, NS, Canada; ^4^Department of Community Health and Epidemiology, Dalhousie University, Halifax, NS, Canada; ^5^Autism Research Centre, IWK Health, Halifax, NS, Canada

**Keywords:** autism, social cognition, moral foundations theory (MFT), morality, punishment, mixed-methods research

## Abstract

Morality can help guide behavior and facilitate relationships. Although moral judgments by autistic people are similar to neurotypical individuals, many researchers argue that subtle differences signify deficits in autistic individuals. Moral foundation theory describes moral judgments in terms of differences rather than deficits. The current research, aimed at assessing autistic individuals’ moral inclinations using Haidt’s framework, was co-designed with autistic community members. Our aim was to describe autistic moral thinking from a strengths-based perspective while acknowledging differences that may pose interpersonal challenges among autistic youth. We assessed 25 autistic and 23 neurotypical children’s moral judgments using the Moral Foundations Questionnaire for Kids. We used semi-structured interviews and qualitative analysis with a subset of participants to describe children’s moral reasoning. Analyses suggested that autistic and neurotypical children make similar judgments about moral transgressions across all five moral foundations. General linear mixed modeling showed that the greatest predictor of recommending punishment was how bad children deemed moral transgressions to be. We also found a trend that autistic children were more likely to recommend punishment for harmless norms violations than were neurotypical children. Future research could use longitudinal methods to understand the development of moral judgments among autistic and neurotypical children.

## Introduction

Autism spectrum disorder (hereafter autism) is a neurodevelopmental condition characterized by differences in social-communication skills and the presence of repetitive or inflexible patterns of behavior or interests (American Psychiatric Association [APA], 2013). The differences in social-communication skills have been attributed to differences in social cognition including commonsense psychology (e.g., Baron-Cohen, 2000 for review), which is the general human tendency to make sense of other people’s actions by attributing psychological states to them (Moore, 2006). Moral psychology is a related aspect of social cognition that involves reflecting on norms for how to treat other people and non-human animals and coexist with them in society.^1^ Moral psychology is often assumed to require typical commonsense psychology skills for its development. Thus, aspects of moral psychology have been theorized by some to be underdeveloped among autistic individuals (e.g., Shoemaker, 2015). In this study we aimed to describe autistic moral thinking from a strengths-based perspective rather than adopting a deficits lens.

Autistic^2^ individuals often have difficulties interacting with others and with forming and maintaining friendships (Howlin et al., 2013). These difficulties are bidirectional (Milton, 2012) and relational (Morrison et al., 2020). To illustrate, just as autistic individuals may struggle to understand social nuances, neurotypical individuals may misinterpret the intentions of autistic individuals (Sheppard et al., 2016). In addition to communication challenges present in interactions between autistic and neurotypical individuals, difficult interpersonal situations faced by autistic individuals could arise in part from differences in moral development and subsequent moral reasoning. Li et al. (2014) found that autistic children aged 6–12 years did not modulate cooperative behavior in response to immoral acts of their partners whereas neurotypical children cooperated less with perceived moral wrongdoers. Autistic children’s failure to modulate behavior in response to others’ moral transgressions may lead neurotypical peers to like autistic children less, making it more difficult for the latter to develop and maintain friendships or possibly contributing to disproportionate rates of bullying victimization among autistic youth (Maïano et al., 2016).

Rationalist accounts of moral development (e.g., Piaget, 1932; Kohlberg, 1969, 1971) suggest that commonsense psychology skills, such as perspective-taking, are required for moral development. Piaget (1932) found that young neurotypical children use reasoning based on rules, whereas older children and adults rely more on others’ intentionality when reasoning about morality. The ability to consider intentions when judging moral culpability has been a key focus in research on morality in autism, wherein subtle differences have often been construed as deficits (for review see Garcia-Molina and Clemente-Estevan, 2019). However, such research has not shown robust connections between commonsense psychology and moral judgments among autistic individuals (for review see Dempsey et al., 2019). As such, autistic individuals appear to use heuristics for making moral judgments that rely less heavily on commonsense psychology than seen in neurotypical individuals, presenting a challenge to the single developmental pathway to moral maturity posited by rationalist accounts.

In contrast to rationalist theories, Haidt (2001) proposes an “intuitionist” model of moral reasoning wherein humans have evolved the capacity to develop moral intuitions in at least five domains or foundations (i.e., authority/respect, care/harm, fairness/reciprocity, in-group/loyalty, and purity/sanctity; Haidt and Joseph, 2008). The authority/respect foundation concerns virtues of obedience and deference; care/harm is related to kindness and care; fairness/reciprocity is linked to justice and trustworthiness, in-group/loyalty is defined by loyalty, patriotism, and self-sacrifice; and purity/sanctity concerns virtues of temperance, piety, and cleanliness (Haidt and Joseph, 2008).

To the authors’ knowledge, only one study has explicitly investigated Moral Foundations Theory holistically among neurotypical children (Peverill, 2020). In this study, 4-year-olds showed moral sensitivity to the authority/respect, care/harm, fairness/reciprocity, and purity/sanctity foundations, but not to the in-group loyalty foundation. Other researchers have provided support for children’s sensitivity to moral transgressions related to Haidt’s (2001) five moral foundations. Concerns for authority/respect have not been studied in infancy but there is evidence that elementary school children prefer peers who obey authority to peers who disobey authority (Martín-Antón et al., 2016). There is a rich literature supporting children’s sensitivity to care/harm violations emerging as early as at 3 months (e.g., Vaish et al., 2010; Hamlin and Wynn, 2011; Kanakogi et al., 2013, 2017). Fairness/reciprocity has also been extensively studied among young children, which has shown that children as young as 16 months anticipate and prefer equal resource allocation (e.g., Geraci and Surian, 2011; Schmidt and Sommerville, 2011). In terms of in-group/loyalty, Jin and Baillargeon (2017) found that 17-month-old infants anticipated that in-group members would help each other but that individuals who did not belong to the same group would not help each other. Finally, research investigating early emergence of the purity/sanctity foundation is limited but suggests that disgust and unnaturalness contribute to children’s moralization of novel acts (Rottman and Kelemen, 2012).

Crucially, Haidt’s theory does not rely on commonsense psychology for the development of moral judgment. This theory could help make sense of moral psychology among autistic individuals. For instance, autistic adults have described their condition as resulting in greater loyalty, honesty, and empathy for other autistic adults and non-human animals than for their neurotypical peers, which could affect their moral foundations profile (Russell et al., 2019). Zalla et al. (2011) found that autistic participants judged disgusting acts as equally morally wrong as harmful acts, in contrast with neurotypical participants who judged disgusting acts as less morally wrong than harmful ones. This difference could suggest greater prioritization of the purity/sanctity foundation among autistic compared with neurotypical participants. Additionally, some studies have shown that autistic youth, compared with neurotypical youth, place a greater emphasis on authority and rules than on abstract principles such as justice (Takeda et al., 2007; Senland and Higgins-D’Alessandro, 2016; Garon et al., 2018). This difference could signify greater emphasis on the authority/respect moral foundation among autistic compared with neurotypical individuals.

Moral reasoning, i.e., justification for one’s moral judgments, differs between autistic and neurotypical children despite similar moral judgments. When asked to justify moral judgments, autistic youth have been found to reiterate moral vignettes (Grant et al., 2005) or provide concrete, less elaborate justifications more often than their neurotypical peers (Shulman et al., 2012; Garcia-Molina et al., 2019). Autistic children’s justifications tend to be more rule-bound and focused on consequences rather than intentions, compared with those provided by neurotypical children (Takeda et al., 2007; Fadda et al., 2016; Garon et al., 2018; Garcia-Molina et al., 2019).

Differences in moral reasoning between autistic and neurotypical individuals despite relatively similar moral judgments could be explained in part by studies suggesting that moral and other forms of reasoning are *post hoc* rationalizations of intuitive judgments (Haidt, 2001; Mercier and Sperber, 2011). Rule- and consequence-oriented moral reasoning in autistic children may therefore indicate *post hoc* rationalizations for moral judgments that may be explained by the relatively concrete thought processes often observed in autism (Hobson, 2012), as opposed to underdeveloped moral intuitions.

Neurotypical children are sensitive to retributive justice from infancy. Hamlin et al. (2011) found that infants as young as 8 months old preferred agents who acted negatively toward antisocial individuals as opposed to agents who acted positively toward antisocial individuals. Geraci and Surian (2021) found that 21-month-old toddlers anticipated punishment for individuals who did not defend an innocent victim whereas they did not anticipate punishment for individuals who did help an innocent victim. Neurotypical toddlers’ expectations of punishment for non-defenders extends to anticipating corporal punishment but seems restricted to when the object being defended (or not) is agentic rather than inanimate (Geraci, 2021). Children aged 3–7 years have shown a tendency to endorse punishment for wrongdoers even at a cost to themselves (Yudkin et al., 2020; Marshall et al., 2021).

Some differences have been found in assigning blame and punishment to transgressors by autistic compared with neurotypical children. To illustrate, Li et al. (2018) found that autistic children aged 6–12 years were more likely to recommend a child be punished for hitting another child than were neurotypical children. Some researchers have found that blame was less tempered by intent among autistic compared with neurotypical children. For example, Salvano-Pardieu et al. (2016) found that autistic adolescents did not differ in their judgments of the level of seriousness of stabbing compared with punching, whereas neurotypical adolescents deemed stabbing as worse than punching. In contrast, Akechi et al. (2018) found that assigning blame did not differ between autistic and neurotypical youth. Rogé and Mullet (2011) found that use of intent in tempering blame judgments increased with age for both autistic and neurotypical participants; it could be that Akechi et al. (2018) did not find group differences due to their sample’s large age span (7–24 years).

To the authors’ knowledge, moral foundations theory has only been investigated in one study of autistic individuals—a qualitative investigation of moral foundations theory among autistic adults (Dempsey et al., 2020). The aim of the current mixed methods study was to investigate moral decision making and reasoning among autistic youth from the perspective of moral foundations theory. Our first hypothesis was that authority/respect and purity/sanctity moral foundations would be endorsed more strongly by autistic than by neurotypical children. Our second hypothesis was that autistic children would be more likely to indicate that children depicted in morality vignettes should be punished, particularly for social norms violations, compared with neurotypical children. We conducted this analysis while controlling for alexithymia traits, i.e., difficulty identifying and describing emotional states (Griffin et al., 2016), as these have been shown to influence moral decision making in autism (Patil et al., 2016). Our third hypothesis was that moral reasoning by autistic children would more often rely on rules and outcomes than among neurotypical children, as assessed in a subset of our participants using qualitative analysis.

## Materials and Methods

### Participants

Ethical approval for this study was granted by the [institution name removed for blind review]’s research ethics board. Parents and children consented and assented to study participation, respectively. Autistic children aged 8–12 years (*n* = 26) were recruited through the [institution name removed for blind review] and community sources. We selected children in this age range because we anticipated they would be cognitively able to complete our moral judgment task while yet pre-adolescent. Autistic children were diagnosed according to DSM-5 diagnostic criteria by specialist teams prior to their recruitment to the research study, most based at a tertiary children’s hospital (81%), following guidelines that recommend the use of the Autism Diagnostic Observation Schedule (Lord et al., 2015) and the Autism Diagnostic Interview—Revised (Lord et al., 1994). Age-comparable neurotypical participants (*n* = 24) were recruited through community sources. One autistic child was not included in the analysis due to low intellectual abilities. One child from the control group was omitted from analysis due to having a diagnosed learning disability. The final numbers for analysis were *n* = 25 autistic and *n* = 23 neurotypical children. Subsamples of autistic and neurotypical children (*n* = 6 each) were invited to participate in semi-structured interviews regarding their responses to the morality vignettes.

Most children were boys (80% of autistic children; 78% of neurotypical children). The remainder were girls (autistic: 16%; neurotypical: 22%) or transgender (autistic: 4%). The groups were similar in age (autistic *M*_*age*_ = 9 years 1 month; *SD*_*age*_ = 11.5 months; neurotypical *M*_*age*_ = 9 years 4 months; *SD*_*age*_ = 14.6 months), *t*(6.58) = 1.76, *p* = 0.46. This was true for the qualitative subsample as well: autistic *M*_*age*_ = 9 years 9 months; *SD*_*age*_ = 17 months; neurotypical *M*_*age*_ = 8 years 8 months; *SD*_*age*_ = 6.8 months), *t*(41.74) = 0.74, *p* = 0.12. Children were predominantly white (72% of autistic children; 83% of neurotypical children). In the autistic group, 4% of children were Hispanic, 8% African Canadian, 8% were African Canadian/Aboriginal Canadian, and 8% were Aboriginal Canadian. In the neurotypical group, 9% of children were South Asian Canadian, 4% were Asian Canadian, and 4% were Aboriginal Canadian. In terms of parent-reported co-occurring conditions, 32% of autistic participants had co-occurring Attention Deficit/Hyperactivity Disorder (ADHD); 28% had a Learning Disability; 20% had an Anxiety Disorder, 4% had Tourette syndrome, and 4% had been diagnosed with Global Developmental Delay (presumably in the preschool years; current intellectual ability assessed by the Wechsler Abbreviated Scale of Intelligence-Second Edition, WASI-II, Wechsler, 2011, was in the average range). No psychiatric or neurodevelopmental diagnoses were reported among the final neurotypical group. Twenty-eight percent of autistic children had pharmaceutical prescriptions; 86% of this subset of children were prescribed psychiatric medications (e.g., stimulants to treat comorbid ADHD), in contrast to no children in the neurotypical group. There were no significant differences between groups in parent-reported economic or social political orientations, in household income, marriage status, employment status, or parental education (see Table 1).

**TABLE 1:** Demographic variables reported by parents of autistic and neurotypical children.

Demographic variable		Diagnosis			
		% ASD	% NT	*X*^2^ (6)	*p*
Family income				8.55	0.2
	<$20,000	4	0		
	$20,000–$39,000	12	0		
	$40,000–$59,000	12	4		
	$60,000–$99,000	40	35		
	$100,000–$139,000	12	9		
	>$140,000	12	39		
	Prefer not to answer	8	13		
Respondent education				8.65	0.19
	Some high school	4	0		
	Completed high school	4	9		
	Some trade/Vocational school	17	4		
	Completed trade/Vocational school	28	22		
	Undergraduate degree	36	26		
	Master’s degree	8	35		
	Doctoral degree	0	4		
	Other/N/A	4	0		
Spouse’s education				12.58	0.05
	Completed high school	5	17		
	Some trade/Vocational school	16	4		
	Completed trade/Vocational school	24	22		
	Undergraduate degree	24	17		
	Master’s degree	12	22		
	Doctoral degree	0	17		
	N/A	20	0		
Stance on social issues				3.92	0.69
	Very liberal	24	30		
	Slightly liberal	8	13		
	Liberal	12	26		
	Moderate	40	22		
	Conservative	4	4		
	Very conservative	4	0		
	Don’t know/NA	8	4		
Stance on economic issues				6.87	0.33
	Very liberal	17	4		
	Slightly liberal	4	4		
	Liberal	20	35		
	Moderate	36	35		
	Conservative	4	13		
	Very conservative	12	0		
	Don’t know/NA	8	9		
				*X*^2^ (1)	
At least one parent with full-time employment		80	96	1.44	0.23
Parents married or common-law		80	100	3.22	0.07

*ASD, Autism Spectrum Disorder; NT, Neurotypical; X^2^, Chi-squared test statistic.*

### Materials

#### Questionnaire Software

The Moral Foundations Questionnaire for Kids (Curtis et al., 2019) was administered using Cedrus^®^ Superlab software (Cedrus Corporation, 2016). All other questionnaires were administered using LimeSurvey© software (Schmitz, 2012).

#### Computers

A MacBook Pro (Retina, 15-inch, Mid 2015) computer was used for all child-completed measures. Parents completed questionnaires on a MacBook Air (13-inch Mid-2013) computer.

### Measures^3^

The internal consistency of questionnaire responses among our sample was assessed using Cronbach’s alphas. We adopted acceptability cut-off scores of α = 0.6 for scales with 10 or fewer items and α = 0.7 for lengthier scales (Loewenthal, 1996). These and all other statistics were calculated using R (R Core Team, 2020) and R Studio (RStudio Team, 2020).

#### Moral Foundations Questionnaire for Kids

(Curtis et al., 2019, see also Peverill, 2020; measure available from authors). On this measure of moral foundations theory, children heard 4 representative vignettes of each of Haidt’s (2001) five moral foundations through a computer speaker while the text and illustrations were presented on a computer screen. Four additional vignettes were items depicting harmless violations of social norms that are typically not considered moral as such (e.g., wearing pajamas to school), for a total of 24 vignettes. Actors in each vignette were matched to the gender of the child completing the measure. After each vignette, children were asked to indicate whether the act was bad. If they answered “yes,” they were asked to rate “how bad is it?” on a 5-point graphical scale of increasingly large, dark red circles. The measure includes a training block to introduce children to the response options and to ensure that they understand the scale. If children indicated the vignette was not bad, their “how bad is it” response was scored as 0. Summary scores for each moral foundation were calculated for each participant as a metric of how bad children considered each transgression to be across each foundation. Next, if children indicated a vignette was bad, they were asked whether the child in the vignette should be punished (“yes”/“no”) for a score of 1 or 0, respectively. If children indicated the action was not bad, their data for this latter response were treated as missing. See Table 2 for a sample vignette from each moral foundation and the additional social norms violation condition. The Moral Foundations Questionnaire for Kids was piloted among children aged 5–12 years with no known developmental disabilities by researchers in the Early Social Development Lab at Dalhousie University (Hartlin et al., 2018). We adapted it for use with autistic children as part of this research program based on feedback from autistic adults. Cronbach’s α = 0.90 for the ‘‘how bad is it’’ scale in the present sample^4^.

**TABLE 2:** Example vignettes from the Moral Foundations Questionnaire for Kids.

Moral foundation	Example vignette text
Authority/Respect	You see a boy/girl calling his parents bad words. Is this bad?
Care/Harm	You see a boy/girl punch another boy/girl in the stomach. Is this bad?
Fairness/Reciprocity	You see a boy/girl taking all of the cookies and leaving none for others. Is this bad?
In-group/Loyalty	You see a boy/girl teach a secret password to people who are not in his club. Is this bad?
Purity/Sanctity	You see a boy/girl loudly burping and farting while eating. Is this bad?
Social norms condition	You see a boy/girl eating his soup with a fork. Is this bad?

*Each vignette is gendered according to the reported gender identity of the child to whom the task is administered.*

#### Moral Reasoning

A subsample of autistic and neurotypical participants (*n* = 6 each) was asked to describe their moral reasoning in the vignettes using a semi-structured interview (see Supplementary Data Sheet 1)^5^.

#### The Wechsler Abbreviated Scale of Intelligence—Second Edition

The WASI-II (Wechsler, 2011) is a standardized test of cognitive abilities with strong internal consistency and good convergent and discriminant validity (Wechsler, 2011). The first version of this abbreviated test has demonstrated good predictive validity of full Wechsler Intelligence Quotient (IQ) scores among autistic youth (Minshew et al., 2005).

#### Autism Spectrum Quotient: Child Version

The child version of the Autism Spectrum Quotient (Auyeung et al., 2008) is a 50-question parent-report measure of children’s autistic traits. Each item is scored from 0 to 3, with greater scores representing higher autistic traits. An example item is: “Enjoys meeting new people” (reverse scored). This measure differentiates groups of autistic from neurotypical children based on autism symptom severity (Auyeung et al., 2008). Its internal consistency is high: α = 0.97 (Auyeung et al., 2008). Internal consistency of the total score in our sample was α = 0.79.

#### Children’s Alexithymia Measure

The Children’s Alexithymia Measure (Way et al., 2010) is a 14-item parent-report instrument designed to assess emergent alexithymic traits among youth. Parents endorse traits observed in their children on a scale from 0 to 3, with higher scores indicating greater alexithymic tendencies. The measure was developed with parents of 220 youth, aged 5–17 years, who had experienced trauma. An example of an item is: “Has difficulty naming his/her positive feelings (such as joy, happiness, excitement).” The measure’s internal consistency is strong (α = 0.92; Way et al., 2010). Several studies have used this measure with parents of autistic youth (e.g., Griffin et al., 2016; Trevisan et al., 2016). In our sample internal consistency was α = 0.93.

#### Alexithymia Questionnaire for Children

The Alexithymia Questionnaire for Children (Rieffe et al., 2006) is a 20-item self-report measure based on the Toronto Alexithymia Scale (Bagby et al., 1994). Children respond to statements regarding emotional awareness using ratings from 0 to 2, with higher scores representing greater alexithymic traits. The measure’s factor structure and predictive validity were established among 740 neurotypical youth aged 9–15 years (Rieffe et al., 2006). An example item is: “It is difficult for me to say how I really feel inside, even to my best friend.” This measure has been used in several studies investigating alexithymic traits among autistic youth (e.g., Costa et al., 2019). Internal consistency of this measure in our sample was α = 0.66. Given this low alpha, we dropped one problematic item^6^ which raised the alpha to 0.73, and conducted analyses using the 19-item version.

#### The Social Desirability Scale-17 (Adapted)

The Social Desirability Scale-17 (Stöber, 2001) is a set of true-false questions designed to assess the degree to which adult participants present themselves in a socially approved manner. It shows convergent validity (*r* = 0.60) with the Lie Scale of the Eysenck Personality Questionnaire (Eysenck and Eysenck, 1991; Stöber, 2001), and has high internal consistency (Cronbach’s α = 0.80; Stöber, 2001). This measure has been used in morality research with autistic young adults (Garon et al., 2018). We adapted the measure for autistic and neurotypical children by simplifying language and reducing the number of items to 10. An example is: “I always admit when I make mistakes” (original version: “I always admit my mistakes openly and face the potential negative consequences”). The revised measure is available from authors upon request. Internal consistency of this measure in our sample was α = 0.60.

#### Demographics Questionnaire

Participants’ parents were asked to provide demographic information regarding their children’s age, sex, gender, race/ethnicity, co-occurring conditions, and medications. Parents were also asked to provide information about their own education, occupation, and family income. We also collected information on parents’ political orientations toward social and economic issues because political thinking has been linked with differential salience of moral foundations in adults (Koleva et al., 2012).

### Procedures

Children (autistic *n* = 12; neurotypical *n* = 11) were assessed in a research laboratory, or in families’ homes (autistic *n* = 12; neurotypical *n* = 12). One autistic child was assessed in a quiet room in a public library because the family was unable to travel to the laboratory and lacked a quiet place at home. Children completed the Moral Foundations Questionnaire for Kids while being video-recorded for purposes not reported here. The WASI-II was administered next, followed by the Social Desirability Scale. Children either read these questions to themselves or had the questions read to them, depending on each child’s stated preference. Parents completed the Autism Spectrum Quotient: Child Version, the Children’s Alexithymia Measure, and the Demographics Questionnaire in a separate room while their children participated. Six children from each group were offered to be interviewed regarding the reasons behind their responses to the Moral Foundations Questionnaire for Kids based on the first author’s assessment of their verbal reasoning skills. The first author conducted all assessments assisted by two trained undergraduates.

### Community Involvement

This study was designed and executed with the direct assistance of autistic adults in a community-based participatory research framework (Jull et al., 2017). Six adult participants from a previous research project (citation removed for blind review) were invited to offer feedback on the Moral Foundations Questionnaire for Kids (Curtis et al., 2019) to make this measure more suitable for autistic youth. Two autistic adults from this group were consulted as community team members during the design and recruitment phases of this study; they were again consulted to assist with interpretation of findings.

## Results

### Preliminary Analyses

#### Descriptive Statistics

Averaged results and *t*-tests comparing groups on the Social Desirability Scale, Children’s Alexithymia Measure, Autism Spectrum Quotient: Child Version, and WASI-II intelligence scales are reported in Table 3 for the entire sample and for the subset included in the qualitative analysis. Note that for these and all family-wise comparisons in the analyses that follow we employed Benjamini-Hochberg corrections for multiple testing (Benjamini and Hochberg, 1995). This method rank orders *p*-values from the analyses, then assigns a new cut-off point based on the formula: (*i*/*m*) * α with *i* corresponding to the *p*-value’s rank, *m* corresponding to the number of comparisons, and α corresponding to the predetermined alpha rate, in this case 5% based on convention. Among the full sample, autistic children had on average higher alexithymia scores and autistic traits, and lower Full-Scale IQ and Verbal Comprehension scores, than neurotypical children. The two groups did not differ in social desirability or in Perceptual Reasoning scores. Autistic children in the qualitative analysis subsample showed higher autism traits but did not differ significantly on this or other independent variables after correcting for multiple comparisons.

**TABLE 3:** Descriptive and inferential statistics for questionnaire and cognitive measures among autistic (*n* = 25) and neurotypical (*n* = 23) children.

	ASD	NT		
			
	*M* (*SD*)	*M* (*SD*)	*t*	*p*
CAM	**15.40 (8.06)**	**7.26 (5.06)**	**0.539*^a^***	**0.0002**
AQC	**19.08 (6.42)**	**15.12 (4.53)**	**2.431**	**0.0192**
AQ-child	**85.52 (18.21)**	**48.57 (12.38)**	**8.275**	**<0.0001**
Social desirability	6.20 (2.33)	5.82 (1.67)	0.643	0.52
FSIQ	**90.04 (13.43)**	**103.91 (7.78)**	**4.42**	**<0.0001**
VCI	**85.08 (12.98)**	**103.22 (10.22)**	**5.402**	**<0.0001**
PRI	97.64 (18.21)	103.48 (9.88)	1.396	0.17

**Qualitative sample characteristics (*N* = 6)**				

	***M (SD)***	***M (SD)***	***t*-score**	***p***

CAM	18.33 (11.45)	10.67 (5.68)	0.394*^a^*	0.17
AQC	14.50 (9.14)	15.17 (4.79)	0.158	0.88
AQ-child	79.33 (24.64)	51.00 (11.28)	2.561	0.04
Social desirability	6.33 (2.58)	6.00 (1.67)	0.265	0.80
FSIQ	97.17 (17.39)	103.33 (5.50)	0.828	0.44
VCI	88.50 (15.06)	104.83 (12.67)	2.033	0.07
PRI	107.67 (26.24)	101.33 (8.21)	0.564	0.59

*ASD, Autism Spectrum Disorder; NT, Neurotypical; CAM, Children’s Alexithymia Measure (parent report; Way et al., 2010); AQC, Alexithymia Questionnaire for Children (child report; Rieffe et al., 2006); AQ-Child, Autism Spectrum Quotient for Children (parent report; Auyeung et al., 2008); FSIQ, Full-Scale Intelligence Quotient; VCI, Verbal Comprehension Index; PRI, Perceptual Reasoning Index. The latter three scales are from the Wechsler Abbreviated Scale of Intelligence—Second Edition (Wechsler, 2011) administered to the child. ^a^Denotes that Wilcoxon signed rank test was used rather than t-test due to skewed data; r effect size is reported. Bolded values represent statistically significant differences after correcting for multiple comparisons.*

### Primary Analyses

#### Hypothesis One

See Table 4 for descriptive statistics for judgments of transgressions in each group for each of the foundations in the Moral Foundations Questionnaire. Note that sensitivity analysis based on this sample’s *N* suggested the study was sufficiently powered to detect medium to large effect sizes for this and the following analysis. To test the first hypothesis that authority/respect and purity/sanctity moral foundations would be endorsed more strongly by autistic than by neurotypical children, due to skewness we calculated two Wilcoxon signed-rank tests and their effect sizes. Contrary to hypothesis one, we found no significant difference between “how bad” authority/respect transgressions were judged by autistic (*M* = 15.92; *SD* = 4.16) and neurotypical (*M* = 15.43; *SD* = 3.16) children, *r* from Wilcoxon signed-rank test = 0.12, *p* = 0.42. Similarly, contrary to our hypothesis, the assessment of purity/sanctity transgressions did not differ between the autistic (*M* = 13.52; *SD* = 5.94) and neurotypical (*M* = 12.91; *SD* = 6.04) groups, *r* from Wilcoxon signed-rank test = 0.07, *p* = 0.60. Note that we were unable to control for the potential effect of Full-Scale IQ on these analyses because we used Wilcoxon signed-rank tests. As a sensitivity check, we completed two analyses of covariance (ANCOVAs) with Full-Scale IQ as a covariate. Consistent with Wilcoxon signed-rank test results, ANCOVAs yielded non-significant results: effect of diagnosis on assessment of authority/respect *F*(1, 45) = 0.03, *p* = 0.87; and purity/sanctity transgressions: *F*(1, 45) = 0.008, *p* = 0.93.

**TABLE 4:** Average “how bad is it” responses and proportion of vignettes for which children recommended punishment, measured using the Moral Foundations Questionnaire for Kids among autistic and neurotypical children.

	How bad is it?				Should they be punished?			
	ASD	NT			ASD	NT		
						
Foundation	*M* (*SD*)	*M* (*SD*)	*r*	*p*	Percentage (%)		*r*	*p*
Authority/Respect	15.9 (4.16)	15.4 (3.16)	0.12	0.42	88.17	89.77	0.03	0.84
Care/Harm	16.9 (3.91)	17.1 (3.10)	0.06	−0.68	89.25	88.89	0.14	0.35
Fairness/Reciprocity	13.2 (5.23)	10.4 (4.28)	0.26	0.07	72.73	56.25	0.21	0.14
In-group/Loyalty	12.1 (4.13)	10.3 (3.47)	0.20	0.16	80.26	72.22	0.06	0.65
Purity/Sanctity	13.5 (5.94)	12.9 (6.04)	0.07	0.60	75.64	62.96	0.04	0.76
Social norms	**8.48 (5.33)**	**4.26 (3.99)**	**0.42**	**<0.01**	**76.79**	**31.58**	**0.48**	**<0.001**

*ASD, Autism Spectrum Disorder; NT, Neurotypical. r = r effect size from Wilcoxon signed-rank test. Bolded values represent statistically significant differences after correcting for multiple comparisons. Punishment scores are represented using the percentage of times children from each group recommended punishment across foundations and the social norms violation condition, but we calculated Wilcoxon signed-rank tests for the summed punishment score for each foundation between groups to avoid violating the assumption of independence of observations.*

#### Hypothesis Two

See Table 4 for the proportion of vignettes across foundations for which children in each group endorsed punishment. Our second hypothesis was evaluated using a general linear mixed model. The dependent variable was the dichotomous endorsement of whether punishment was recommended. Level one of each model was the repeated measures within-subjects factor. Level two predictors were between-subjects autism diagnosis, children’s “how bad is it” ratings, and their interaction, while controlling for alexithymia (by both child and parent report) and Full-Scale IQ. The only effect that remained significant after Benjamini-Hochberg correction was the main effect of “how bad is it” responses: *OR* = 2.58; 95% CI = [2.00, 3.33], *p* < 0.001, suggesting that, across groups, more severe judgments of the vignettes were associated with greater probability of endorsing punishment for transgressions. We describe other effects that would have been significant without corrections in terms of trends. There was only one such trending effect, of diagnostic group: *OR* = 0.16; 95% CI = [0.03, 0.82], *p* = 0.028. Given that this odds ratio is less than 1, we have taken its reciprocal for ease of interpretation: 1/0.16 = 6.25 meaning that autistic children were 6.25 times more likely than neurotypical children to endorse punishment for transgressions. An *a priori* planned comparison suggested that, consistent with the hypothesis, this group trend was driven by significantly more endorsement of punishment in the social norms violation condition by autistic than neurotypical children, *r* from Wilcoxon signed-rank test = 0.48, *p* < 0.001. Marginal *R*^2^ and Conditional *R*^2^ were 0.31 and 0.55, respectively.

#### Hypothesis Three

Qualitative data relating to the third hypothesis were analyzed using reflexive thematic analysis (Braun and Clarke, 2006, 2019). We used semantic coding from a realist perspective with a mix of inductive and deductive coding. Statements, i.e., “phrases … which retained a sense of completeness and had an [*sic*] homogeneous object” (Sani and Reicher, 1998) could be coded multiple times if they fit into more than one theme. After reading all transcribed interviews, the first author coded the data inductively and generated initial themes. She then focused on themes relevant to moral foundations theory to address the research questions. To discern whether children’s rationales fell within one of Haidt’s (2001) moral foundations, the coding scheme developed by the first author drew on research and theory of moral foundations theory (Haidt, 2003; Haidt and Joseph, 2008; Graham et al., 2011; Clifford et al., 2015). Some transgressions were deemed bad for multiple reasons, so statements could be coded under more than our theme. Next, an experienced Master’s-trained research staff member familiar with the underlying theory performed analytic auditing on the coded data, ensuring that data fit into the identified themes without redundancy (Elliott and Timulak, 2005). Where codes did not seem to fit within themes, the two coders discussed the discrepancy and altered coding as necessary. Themes were divided by whether children judged actions depicted in the vignettes as bad or not bad by their reports. To remain consistent with qualitative methodology, frequencies of coded extracts in support of themes for the two groups were not compared statistically.

##### Bad

**FIGURE 1:**
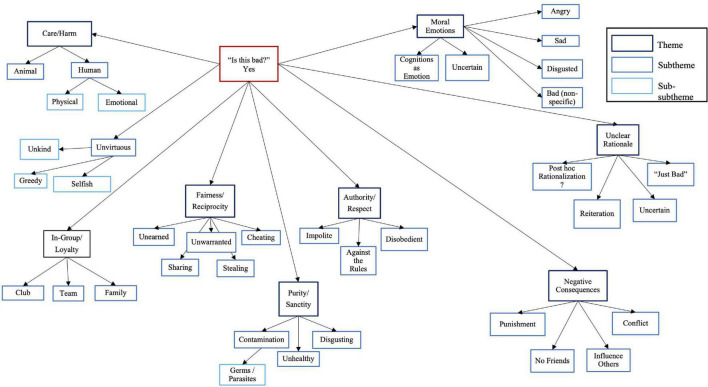
Coding tree for qualitative analysis when autistic and neurotypical children judged moral vignettes to be “bad.”

All five foundations of moral foundations theory were represented among autistic and neurotypical children’s justifications for their judgments of actions as morally bad (see Figure 1 for coding tree and Supplementary Table 1 for quotes supporting themes). Some rationales were unclear, i.e., when it wasn’t clear to coders what the rationale was or how it related to the act in the vignette (subthemes: Just Bad, *Post Hoc* Rationalization, Reiteration, Uncertain). The theme of *Post Hoc* Rationalization was supported by rationales that were elaborate but did not seem to make sense given the vignette, e.g., the notion that a child wearing pajamas to school was bad because it might lead to a flea infestation when she returned home.

“Bad” themes were quite equally represented between the two groups including those relating to rules and outcomes (contrary to hypothesis three). One exception was Moral Emotions (Haidt, 2003): autistic children reported feeling “sad” four times more often than did neurotypical children. Further, autistic children indicated uncertainty regarding emotions four times more often than did neurotypical children.

##### Not Bad

**FIGURE 2:**
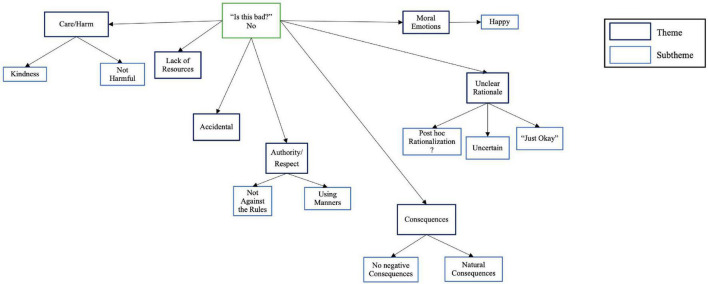
Coding tree for qualitative analysis when autistic and neurotypical children judged moral vignettes to be “not bad.”

See Figure 2 for coding tree and Supplementary Table 2 for quotes in support of themes. The Not Harmful subtheme, i.e., when children indicated an act was not bad because no one was harmed, was more represented among autistic than among neurotypical participants. Contrary to our third hypothesis, Consequences (e.g., no punishment is required for the act) were alluded to more often among neurotypical than among autistic children. As with the bad vignettes, an Unclear Rationale theme emerged. The subtheme Just Okay referenced instances when children did not provide further elaboration for their moral decisions. This theme was more evident among autistic than neurotypical children, whereas more neurotypical children offered *post hoc* rationalizations to justify their “not bad” judgments. A theme of Positive Emotions when children deemed acts to be not bad, i.e., when they indicated feeling happy or that the vignette was humorous, was evident more often among autistic than neurotypical children.

## Discussion

This was the first investigation of moral reasoning and judgment among autistic children from the perspective of moral foundations theory (Haidt, 2001). Our aim was to begin to develop an understanding of similarities and differences in moral foundation priorities between autistic and neurotypical children, to investigate recommendations for punishments for moral transgressions between the two groups, and to describe the children’s moral reasoning.

Our first hypothesis, that autistic children would be more disapproving of authority/respect and purity/sanctity foundations violations than neurotypical children, was not supported. Other studies have shown that older autistic individuals may be more sensitive to violations of these foundations than neurotypical adults (Zalla et al., 2011; Senland and Higgins-D’Alessandro, 2016). Perhaps differences in judgments regarding purity/sanctity among autistic individuals do not emerge until adolescence or adulthood. An alternative explanation for our divergent results is that our study employed more scenarios for each form of transgression (four versus two each in the Zalla et al., 2011, study). Perhaps this generated more representative responses leading to similar judgments of the foundations for both groups.

In terms of lack of differences in the authority/respect foundation judgments, past research has assessed moral values based on Kohlberg’s 1969, 1971 stage-based developmental moral hierarchy (Takeda et al., 2007; Senland and Higgins-D’Alessandro, 2016; Garon et al., 2018). This theoretical framework leads to interpretations based on the stage of moral maturity at which the child’s moral reasoning is deemed to lie. In contrast, we asked children to rate how bad each transgression was across all five moral foundations and without setting up a hierarchy. This methodological difference could have affected our results. Alternatively, our scale may have contributed to a ceiling effect. Indeed, 104 authority question responses reached the maximum “how bad is it” score. Autistic children may have indicated significantly more disapproval of authority transgressions had our scale permitted a broader range of responses.

Our second hypothesis that autistic children would be more likely than neurotypical children to recommend punishment for harmless social norms violations was partially supported, consistent with past findings (Rogé and Mullet, 2011; Salvano-Pardieu et al., 2016; Li et al., 2018). This could be due in part to the social communications differences observed among autistic children (American Psychiatric Association [APA], 2013). It is possible that they are more prone to making harmless social norms violations than their neurotypical peers and may experience correction or even punishment for these differences. Autistic children may in turn assume that other children who behave similarly will or should be punished for such behavior. It is important to note that the judgment of how bad the transgression was deemed by participants was a stronger predictor of endorsing punishment than was being autistic. Indeed, the effect of diagnostic group was only trending as significant in the general linear mixed model (i.e., only significant prior to correction for multiple testing).

Our third hypothesis, that autistic children’s moral reasoning would defer to rules and consequences more often than that of neurotypical children, was not supported—autistic children adduced these reasons *less* frequently than neurotypical children. This result, which is inconsistent with past research (Fadda et al., 2016; Garon et al., 2018; Garcia-Molina et al., 2019), could be due to differences in coding methodology. Most studies assessing autistic children’s moral reasoning have used pre-specified coding schemes designed to contrast consideration of intentionality with more concrete reasoning. Our inductive thematic analysis may have yielded more diverse themes without forcing children’s responses into pre-existing categories.

Consistent with past research (Grant et al., 2005; Shulman et al., 2012), autistic children sometimes offered responses that were less elaborate than those of neurotypical children; that is, they more often said that acts were “just okay” without elaboration. However, this difference was not apparent when children were asked to justify their judgments that actions in the vignettes were bad. Interestingly, neurotypical children’s responses to the vignettes were sometimes more elaborate, but more often made little sense. For instance, when asked why it would be bad to wear pajamas to school, one neurotypical child reasoned that one might need to swim in them, which would be uncomfortable. Coders judged these explanations to be *post hoc* rationalizations. Given the theorized social element of *post hoc* rationalizations (Haidt, 2001; Mercier and Sperber, 2011), perhaps the autistic children’s lesser use of such justifications reflects their social communication differences (American Psychiatric Association [APA], 2013). Alternatively, Grant et al. (2005) found that appropriate justifications for moral judgments were positively correlated with age among autistic and neurotypical children. The neurotypical children were younger than the autistic children in our subsample, although this difference did not reach statistical significance. Still, this age difference could have contributed to neurotypical children’s greater propensity to offer these types of justifications. A further possible explanation for the finding that autistic children provided less elaborate responses than the neurotypical children could be the lower verbal cognitive abilities among the former group.

A further finding of our qualitative analysis is that autistic children reported sadness (e.g., in response to a vignette depicting a child eating soup with a fork rather than a spoon), as well as uncertainty regarding their emotions more often than did their neurotypical peers. This could be due in part to higher rates of alexithymia among the autistic children in our sample, consistent with past research (Hill et al., 2004).

In general, our results suggest that judgments regarding transgressions against moral foundations made by neurotypical and autistic children were more similar than different, despite differences found in previous research (e.g., Garon et al., 2018), and despite markedly lower verbal reasoning skills among the autistic children. An explanation is that differences in social cognition in autism could present as differences in moral values when assessed based on rationalist theories, but that differences in valuing of the five moral foundations are not apparent when studied explicitly, as we did. To illustrate, autistic children have been found to focus more on rules and consequences than neurotypical children when judging the severity of moral transgressions (e.g., Fadda et al., 2016). In our Introduction, we interpreted such differences as possibly reflecting variations in valuing of the authority/respect foundation among autistic compared with neurotypical children. We argue that such differences may instead reflect relatively concrete (Minshew et al., 2002) or rigid (Poljac and Bekkering, 2012) thinking in autism without denoting higher prioritization of authority/respect over other moral foundations.

This interpretation, coupled with differences in verbal reasoning between groups, is consistent with our suggestion that moral psychology may develop through a pathway other than the commonsense psychology route suggested by rationalist theories. This interpretation also fits with results from Dempsey et al. (2020) that the autistic adults in their study, who were generally politically left-wing, emphasized the importance of care/harm and fairness/reciprocity above the other moral foundations posited by Haidt (2001). This profile of moral foundations has been observed to differentiate politically left- from right-wing ideologies (Graham et al., 2009). Given the homogeneity of political attitudes espoused by the parents of children in the current study, it is possible that broader cultural influences may be more responsible for the development of sensitivity to moral transgressions across foundations than are differences in social cognition in autism. However, given the novelty of the present study, and the fact that little research exists on the development of moral foundations theory even among neurotypical children (see Peverill, 2020), this interpretation is speculative. Resolving this issue would require testing with more politically heterogeneous groups of families of autistic and neurotypical children.

An interesting avenue for further exploration of moral foundations theory in autistic and neurotypical youth would be to investigate whether differing profiles of moral foundations predilections are present between the two groups. Researchers could add gender to these analyses in a larger sample in future to investigate the role of gender on moral decision making among autism and neurotypical individuals. This type of profile analysis has been conducted in studies with larger, more politically and culturally heterogeneous samples (e.g., Graham et al., 2009; Vecina and Chacón, 2019). Future research should also clarify whether autistic children are equally likely as neurotypical children to anticipate punishment for agents who have not defended an innocent victim in accordance with the care/harm foundation of moral foundations theory (Haidt, 2001; Geraci, 2021; Geraci and Surian, 2021).

Understanding the developmental course of moral foundations predilections, severity of judgments, and recommendations for punishment could offer guidance for interventions for autistic and neurotypical children to promote clarity and improve communication among children on the continuum from autistic to neurotypical. Such an intervention could take the form of a social narrative (Gray and Garand, 1993) for autistic and neurotypical children that illustrates the unique developmental challenges faced by some autistic children viz. punishment and morality. Another possibility would be a psychoeducational intervention for parents and educators illustrating the potential costs of applying negative consequences for harmless norms violations among autistic children, though additional research would be required first to validate this connection, then to test the intervention.

### Limitations

As do all studies, ours has limitations. First, there were differences in intellectual abilities between the two groups, with neurotypical children showing higher average Full-Scale IQ and Verbal Comprehension scores than the autistic children. These differences did not reach significance among the subsample of children who participated in our qualitative analysis, perhaps due to small number of participants rendering the significance tests insufficiently powered to detect small between-groups differences. Relatively few participants is a general limitation of this study as it may have been underpowered to detect other potential smaller magnitude between-group differences. We controlled for differences in Full-Scale IQ in our quantitative analyses, but were unable to do so for our qualitative analysis. Matching groups of neurotypical and autistic children on IQ is challenging due to the uneven profiles of cognitive strengths and weaknesses documented among autistic individuals (Coolican et al., 2008). The attempt to match groups on age and IQ has led many studies to include only autistic individuals with at least average IQ (Mottron, 2004). Therefore, though IQ differs between the two groups, a strength of our study is that it includes perspectives of autistic children with a range of intellectual abilities. Another limitation is that, for the qualitative component of the study, the autistic children were older than the neurotypical children, which could have confounded our results (although this age difference did not reach statistical significance). A further limitation is that the first author selected the participants for interview based on a clinical judgment of each child’s verbal ability. As such, the selection could have been biased in unknown ways and may not represent the population from which our sample was drawn. Despite these limitations, our results contribute substantially to the literature by offering an initial analysis of moral foundations theory (Haidt, 2001) among autistic and neurotypical children.

## Conclusion

Overall, our quantitative and qualitative findings suggest that autistic and neurotypical children evaluate moral transgressions across Haidt’s (2001) five moral foundations similarly, despite previous research suggesting possible group differences in the relative salience of the authority/respect and purity/sanctity foundations. This was the case despite some differences in their moral reasoning and in the emotions elicited in response to the scenarios we presented. The most prominent difference that emerged from our study was autistic children’s greater likelihood of recommending punishment for relatively minor transgressions.

Insufficient understanding of autistic individuals has been cited by members of the autism community as a barrier to fitting into society (Pellicano et al., 2014). As such, autistic individuals and stakeholders (e.g., families of autistic people) have called for researchers to focus on topics that affect autistic individuals’ day-to-day lives (Pellicano et al., 2014). Our study was the first to investigate moral foundations theory in autistic and neurotypical children. Our findings that autistic children’s moral reasoning differs only subtly from that of neurotypical children contributes to our understanding of moral agency in autism and challenges assertions by some that autistic individuals have limited moral agency (e.g., Richman, 2018). Future studies could use longitudinal methods to track the development of moral foundations predilections and recommendations for punishment to further refine our understanding of moral development in typical development and in autism.

## Data Availability Statement

The raw data supporting the conclusions of this article will be made available by the authors, without undue reservation.

## Ethics Statement

The studies involving human participants were reviewed and approved by the Research Ethics Board of the IWK Health Centre, Halifax, Nova Scotia, Canada. Written informed consent to participate in this study was provided by the participants’ legal guardian/next of kin.

## Author Contributions

ED conceptualized the project, collected and interpreted data, and wrote the manuscript. CM, SJ, SS, and IS contributed equally to collaborating with ED to conceptualize the research project, interpret data, and revise the manuscript. All authors contributed to the article and approved the submitted version.

## Conflict of Interest

The authors declare that the research was conducted in the absence of any commercial or financial relationships that could be construed as a potential conflict of interest.

## Publisher’s Note

All claims expressed in this article are solely those of the authors and do not necessarily represent those of their affiliated organizations, or those of the publisher, the editors and the reviewers. Any product that may be evaluated in this article, or claim that may be made by its manufacturer, is not guaranteed or endorsed by the publisher.

## Footnotes

1 Note, however, that some behavior that is driven by moral convictions may lead to social conflict (e.g., Hauser, 2012; Mcloughlin, 2018).

2 We use identity-first language (i.e., “autistic”; “neurotypical”) rather than person-centered language (i.e., “person with autism”; “typically developing person”), as preferred by many autistic individuals (Kenny et al., 2016).

3 Note that we collected psychophysiological data from a subset of participants but these data are not reported here.

4 Note that, due to the high proportion of missing data for the punishment question (19%), we were unable to calculate alpha for this scale. The option of scoring punishment as 0 for vignettes which children deemed “not bad” was considered. However, given that doing so would zero-inflate the data, thereby overestimating the alpha and potentially confounding results, we opted not to take this option.

5 Note that all participants (and their parents) who were invited to participate in the interview agreed and consented to participation.

6 The item dropped from our analysis was: “Thinking about how I feel helps me when I want to do something about my problems.”
