# Supplementary material for: Moral Foundations Theory Among Autistic and Neurotypical Children
Source: Front Psychol. 2022 Jan 14;12:782610. doi: 10.3389/fpsyg.2021.782610 (PMC8795511; doi:10.3389/fpsyg.2021.782610)
Supplement: Supplementary file 1 [file Data_Sheet_1.docx]

**Appendix**

**Interview Regarding Moral Foundations Questionnaire Responses**

*First off, could you please list five things that you think are morally wrong?*

*What about five things that are morally right?*

The interviewer will then review each participants’ Moral Foundations Questionnaire for Kids responses. For all vignettes the interviewer will reiterate the vignette supplemented by the cartoon drawing accompanying the vignette, and ask:

- - *Why was it wrong/okay?*
  - *How did it make you feel?*
