# Supplementary material for: Moral Foundations Theory Among Autistic and Neurotypical Children
Source: Front Psychol. 2022 Jan 14;12:782610. doi: 10.3389/fpsyg.2021.782610 (PMC8795511; doi:10.3389/fpsyg.2021.782610)
Supplement: Supplementary file 2 [file Table_1.DOCX]

Supplementary Table 1

Quotes and frequency of quotes in support of themes when autistic and neurotypical participants deemed moral transgressions and norms violations to be “bad”

|  |  | Sub-sub-theme |  |  | Frequency | |
| --- | --- | --- | --- | --- | --- | --- |
| Theme | Sub-theme |  | Quote | Diagnosis | ASD | NT |
| Authority/Respect |  |  |  |  |  |  |
|  | Against the Rules |  | I: Why is it bad if it’s not pajama day?  R: Well, you’re not following the rules—the rules are don’t wear pajamas to school if it’s not pajama day. | ASD | 18 | 18 |
|  | Disobedient |  | I: You see a boy ignore his parents when they tell him to stop watching TV. Why is that wrong?  R: Number one, he’s not being obedient. | ASD | 3 | 11 |
|  | Impolite |  | I: You see a boy loudly burping and farting while eating. Why is that bad?  R: It’s not polite. | ASD | 6 | 10 |
| Care/Harm |  |  |  |  |  |  |
|  | Harm to Animals |  | I: You see a girl stomp on the tail of her pet cat. Why is that wrong?  R: Oh, I love cats and dogs. It’s bad cause it’s trying to hurt animals and trying to kill them. | ASD | 5 | 10 |
|  | Harm to Humans |  |  |  |  |  |
|  |  | Emotional | I: You see a boy calling his parents bad words. Why is that bad?  R: It would hurt your parents’ feelings. | ASD | 24 | 25 |
|  |  | Physical | I: You see a boy punch another boy in the stomach. Why is that wrong?  R: Because, it could stop their breathing or something like that. | NT | 6 | 8 |
|  | Unvirtuous |  |  |  |  |  |
|  |  | Greedy | I: You see a boy cut to the front of the line. Why is that bad?  R: Sometimes people wait there for like days, and then he just cuts in and takes, like, five minutes to get something that he wants and that’s just being greedy. | NT | 1 | 4 |
|  |  | Selfish | I: You see a girl cheating in a board game. Why is that bad?  R: Because it’s rude and selfish for her to win. | NT | 0 | 3 |
|  | Unvirtuous |  |  |  |  |  |
|  |  | Unkind | I: You see a boy calling a boy stupid. Why is that bad?  R: Because it’s a bad word and it’s being mean to someone. | NT | 24 | 20 |
| Fairness/ Reciprocity |  |  |  |  |  |  |
|  | Cheating |  | I: You see a boy score a goal against his own team to help the other team win. Why was that bad?  R: Because … he would probably be cheating if he did that. | NT | 6 | 7 |
|  | Sharing |  | I: You see a boy taking all of the cookies, and leaving none for others. Why is that wrong?  R: Because, you’re supposed to share. | NT | 1 | 5 |
|  | Stealing |  | I: You see a boy taking all of the cookies, and leaving none for others. Why is that bad?  R: Because, someone else might have made it and might paid for it and it might be a special day but he stoled [sic] all the cookies. | ASD | 3 | 1 |
|  |  |  |  |  |  |  |
|  | Unearned |  | I: You see a boy cheating in a race by taking a shortcut. Why is cheating bad, I wonder, what do you think?  R: Because you shouldn’t have won, but you did.  I: And why is that bad?  R: Because he didn’t deserve to. | ASD | 10 | 12 |
|  | Unwarranted |  | I: You see a boy punch another boy in the stomach. Why is that bad?  R: Cause … what if the other guy didn’t do anything and he was just trying to be nice and what if the other boy just punched him in the stomach and then the other guy gets hurt. | NT | 1 | 3 |
| In-Group/ Loyalty |  |  |  |  |  |  |
|  | To Club |  | I: You see a boy teach a secret password to people who are not in his club. Why is that bad?  R: Because they’ve got to be in the club. | NT | 1 | 2 |
|  | To Family |  | I: You see a boy reading his brother’s secret diary. Why is that bad?  R: Because it was his brothers’ secret  I: And why is it bad to read someone’s secrets like that, I wonder?  R: Because, it’s being mean to your siblings. | NT | 0 | 6 |
|  | To Team |  | I: You see a boy score a goal against his own team to help the other team win. Why is that wrong?  R: That’s betraying your team basically. | ASD | 3 | 2 |
| Purity/ Sanctity |  |  |  |  |  |  |
|  | Contamination |  | I: You see a girl rubbing poop on herself in the shower. Why is that bad?  R: Well, same for the diaper one, it’s bad for your personal hygiene. | ASD | 12 | 9 |
|  | Germs/Parasites |  | I: You see a boy rubbing poop on himself in the shower. Why is that bad?  R: Because you’re gonna get dirty.  I: Why is it bad to get dirty?  R: Because it’s germy. | ASD | 5 | 1 |
|  | Disgusting |  | I: You see a boy loudly burping and farting while eating. Why is that bad?  R: Because, it would be really gross and stinky. | NT | 5 | 5 |
|  | Unhealthy |  | I: You see a boy drinking pee with his dinner. Why is that bad?  R: Because you could get sick and die. | NT | 5 | 6 |
| Negative Consequences |  |  |  |  |  |  |
|  | Create Conflict |  | I: You see a boy cut to the front of the line. Why is that bad? R: There would be a lot of arguing, and it would start, like, a fight, or something. | NT | 1 | 2 |
|  | No Friends |  | I: You see a boy punch another boy in the stomach. Why is that bad?  R: Because it would hurt the other boy, and, uhm, it could start a fight with him and them punching him back. And they’ll not be friends anymore. | NT | 3 | 6 |
|  | Others might do it too |  | I: I: You see a boy cut to the front of the line. Why is that bad?  R: Cause it’s butting, and you’re supposed to wait at the end of the line and wait for your turn.  I: What’s wrong with not waiting your turn?  R: Because people might start to do it more. | NT | 1 | 3 |
|  | Punishment |  | I: You see a girl calling her teacher bad words. Why is that bad?  R: Well, that could have hurt the teacher’s feelings. And, she could have to go to the office and be in trouble. And go home, and get punished. | ASD | 11 | 14 |
| Unclear  Rationale |  |  |  |  |  |  |
|  | Just Bad |  | I: You see a boy punch another boy in the stomach. Why is that bad?  R: Because, it’s bad. | ASD | 3 | 1 |
|  | Post hoc |  | I: You see a girl wearing her pajamas to school instead of wearing normal clothes. Why is that bad?  R: Because, fleas might get on her and attach to her, and come home and then she’ll have fleas in her hair, and then it will get on her parents, her cat, and then they’ll all have to get scrubbed. | ASD | 6 | 8 |
|  | Reiteration |  | I: You see a boy ignore his parents when they tell him to stop watching TV. Why is that bad?  R: Because he is ignoring his parents. | ASD | 3 | 0 |
|  | Uncertain |  | I: You see a boy ignore his parents when they tell him to stop watching TV. What’s bad about that?  R: Because, he’s supposed to listen to his parents, and you shouldn’t watch TV.  I: Why is it bad not to listen to your parents?  R: I don’t know. | NT | 12 | 9 |
| Moral  Emotions |  |  |  |  |  |  |
|  | Angry |  | I: How does that make you feel? How did you feel towards that boy who kicked the dog?  R: Mad. | ASD | 53 | 49 |
|  | Bad (non-specific) |  | I: How do you feel towards the girl who told the secrets?  R: Negative. | NT | 3 | 7 |
|  | Disgusted |  | I: How does [a boy rubbing poop on himself in the shower] make you feel?  R: Disgusted. | ASD | 12 | 12 |
|  | Sad |  | I: How did [a girl eating her soup with a fork] make you feel?  R: Just a little sad. | ASD | 29 | 7 |
| Reasons as Emotions |  |  | I: How does that make you feel?  R: I feel like why am I even friends with a person that just lies, that like, doesn’t even talk to me that much. | NT | 3 | 1 |
| Uncertain Re: Emotion |  |  | I: How does that make you feel?  R: I don’t know. | ASD | 20 | 5 |

*Note*. ASD: autism spectrum disorder; NT: neurotypical; I: interviewer; R: respondent
