# Supplementary material for: Moral Foundations Theory Among Autistic and Neurotypical Children
Source: Front Psychol. 2022 Jan 14;12:782610. doi: 10.3389/fpsyg.2021.782610 (PMC8795511; doi:10.3389/fpsyg.2021.782610)
Supplement: Supplementary file 3 [file Table_2.DOCX]

Supplementary Table 2

Quotes in support of themes when autistic and neurotypical participants deemed moral transgressions and norms violations to be “not bad”

|  |  |  |  | Frequency | |
| --- | --- | --- | --- | --- | --- |
| Theme | Sub-theme | Quote | Diagnosis | ASD | NT |
| Authority/Respect |  |  |  |  |  |
|  | Not Against  the Rules | I: You see a girl wearing her pajamas to school instead of wearing normal clothes. Why is that bad?  R: It actually could be good too, because if it is pajama day then it would be good. | ASD | 2 | 3 |
|  | Use Manners | I: You see a boy loudly burping and farting while eating. Why isn’t that bad?  R: Cause he might say “excuse me,” and he can’t help burping and farting while he’s eating. | NT | 10 | 1 |
| Care/Harm |  |  |  |  |  |
|  | Kindness | I: You see a boy score a goal against his own team to help the other team win. You said that’s not bad.  R: Because, you’re being nice to the other person. | ASD | 3 | 5 |
|  | Not Harmful | I: You see a boy drinking pee with his dinner. Why is that not wrong?  R: It’s not wrong because it wasn’t harming anyone. | ASD | 1 | 1 |
| Consequences |  |  |  |  |  |
|  | Natural  Consequences | I: You see a boy cheating in a race by taking a shortcut. Why is that okay?  R: Cause, you just get eliminated. | NT | 1 | 3 |
|  | No Negative  Consequences | I: When you saw a girl eating her soup with a fork you said that’s not bad. R: It’s not like you need to get punished for doing something you like. | ASD | 1 | 5 |
| Unclear  Rationale |  |  |  |  |  |
|  | Just Okay | I: You see a boy eating his soup with a fork. Why is that okay?  R: Because, it’s okay. | ASD | 15 | 7 |
|  | Post Hoc | I: You see a girl using a dirty diaper as a pillow. Why is that okay?  R: Well, because it’s her choice, and probably she had no pillow and she just had to do it on her little sister’s or her little brother’s diaper. | NT | 1 | 10 |
|  | Uncertain | I: You see a boy eating his soup with a fork. Why is that okay?  R: It’s his idea.  I: and why does that make it okay?  R: because… I don’t really know. | NT | 1 | 3 |
| Accidental |  | I: You see a boy score a goal against his own team to help the other team win. Why is that okay?  R: Cause, maybe, he did it by accident. | NT | 2 | 6 |
| Lack of Resources |  | I: You see a girl eating her soup with a fork. Why is that okay?  R: Because sometimes my mom and dad don’t give me a spoon. | NT | 0 | 4 |
| Positive Emotions |  |  |  |  |  |
|  | Happy | I: You see a boy score a goal against his own team to help the other team win. Why is that okay?  R: Because, it helped them win the game and sometimes you can be nice to help other people.  I: How does that make you feel?  R: Kind of makes me feel happy to help other people. | NT | 5 | 1 |

*Note*. ASD: autism spectrum disorder; NT: neurotypical; I: interviewer; R: respondent
